# The Conventional Gait Model’s sensitivity to lower-limb marker placement

**DOI:** 10.1038/s41598-022-18546-5

**Published:** 2022-08-20

**Authors:** M. Fonseca, M. Bergere, J. Candido, F. Leboeuf, R. Dumas, S. Armand

**Affiliations:** 1 Kinesiology Laboratory, Geneva University Hospitals and University of Geneva, Geneva, Switzerland; 2 Univ Lyon, Univ Gustave Eiffel, Université Claude Bernard Lyon 1, LBMC UMR_T 9406, 69622 Lyon, France; 3 University of Geneva and University of Applied Sciences and Arts Western Switzerland, Geneva, Switzerland; 4 Centre Hospitalier Universitaire de Nantes, Nantes, France; 5 School of Health and Society, The University of Salford, Salford, UK

**Keywords:** Motor control, Pathology, Physical examination, Paediatric research, Biomedical engineering, Computational science

## Abstract

Clinical gait analysis supports treatment decisions for patients with motor disorders. Measurement reproducibility is affected by extrinsic errors such as marker misplacement—considered the main factor in gait analysis variability. However, how marker placement affects output kinematics is not completely understood. The present study aimed to evaluate the Conventional Gait Model’s sensitivity to marker placement. Using a dataset of kinematics for 20 children, eight lower-limb markers were virtually displaced by 10 mm in all four planes, and all the displacement combinations were recalculated. Root-mean-square deviation angles were calculated for each simulation with respect to the original kinematics. The marker movements with the greatest impact were for the femoral and tibial wands together with the lateral femoral epicondyle marker when displaced in the anterior–posterior axis. When displaced alone, the femoral wand was responsible for a deviation of 7.3° (± 1.8°) in hip rotation. Transversal plane measurements were affected most, with around 40% of simulations resulting in an effect greater than the acceptable limit of 5°. This study also provided insight into which markers need to be placed very carefully to obtain more reliable gait data.

## Introduction

Three-dimensional gait analysis provides large amounts of information used to characterize motor disorders such as cerebral palsy (CP) and plays a demonstrated important role in supporting treatment decision-making^1,2^. Reflective markers are attached to specific anatomical landmarks and used to build a biomechanical model for calculating patients’ kinematics (anatomical segment motion with respect to the ground and other segments). One of the most used models in clinical practice is the Conventional Gait Model (CGM), also known as the Plug-in Gait model^3,4^. The CGM defines lower-limb geometry via a set of seven anatomical segments and a hierarchical top-down process^5^. The kinematic data are calculated from marker trajectories on a frame by frame basis^5^. Any measurement errors in gait analysis introduce variability into the output data and negatively impact data interpretation^6^. Marker placement has been reported as the primary cause of variability in gait analysis^7,8^. Because of the CGM’s process, wrongly placed markers will affect the definition of segment lengths and thus how far all other segments are from them.

Many studies have quantified the general variability caused by gait analysis marker placement by repeating measurements under identical conditions with either the same or different examiners^8^. Based on their results, 2° and 5° were defined as the optimal and acceptable thresholds for measurement differences, respectively. Moreover, the transversal plane was found to be the most sensitive to marker placement. For instance, hip joint rotation results have been reported to have a variability above acceptable limits (5°), and they should be considered with extreme caution^8^. Gait scores, such as the Gait Profile Score (GPS) or the Gait Deviation Index, are also used to evaluate motor disorders by providing an overall gait score with respect to reference asymptomatic data^9,10^. As gait scores are calculated based on kinematics, they are also expected to be sensitive to marker placement. Estimating the expected errors resulting from marker misplacement is therefore important, as is its impact on both kinematic data and gait scores. To the best of our knowledge, gait score sensitivity to marker misplacement has never before been addressed.

One study has evaluated the precision with which investigators place markers^11^. It reported average pelvic and lower marker placement errors of 6–21 mm and 13–25 mm for intra- and inter-examiner, respectively. *Mcfadden *et al. demonstrated that the CGM was more sensitive to poorly placed thigh, knee, and tibia markers in anterior–posterior movements^12^. Another study evaluated the impact of different lateral femoral epicondyle marker placements on kinematics, and it reported differences of up to 5.3° per 10 mm of marker displacement in the anterior–posterior axis^13^. However, little information is available concerning the sensitivity of kinematics to the placement of the complete set of CGM markers.

In practice, the variability resulting from imprecise marker placement is due to the combined imprecision of the placement of all markers together. Thus, this study aimed to evaluate the CGM’s sensitivity to overall lower-limb marker placement. To do so, we simulated marker displacements over the results from CGM measurements made using its basic marker-set configuration. This study was an extension of our previous sensitivity analysis focusing on the knee’s lateral epicondyle marker^13^.

## Methods

### Data collection

Original gait data were collected retrospectively from 20 children: 10 children with CP (6 males and 4 females, mean (standard deviation): age, 12.4 (4.7) years old; height, 150.0 (22.7) cm; and weight, 45.1 (26.4) kg), at Gross Motor Function Classification System levels I and II (five bilateral and five unilateral), and 10 typically developing children (TDC) (8 males and 2 females, mean (standard deviation): age, 13.7 (3.2) years; height, 160.8 (19.1) cm, and weight, 49.5 (17.7) kg). After anatomical palpation, markers were placed according to the guidelines by an investigator with over 10 years of continuous practice experience^14^. All methods carried out in this study were in accordance with the guidelines for gait analysis in clinical practice. This study was approved by the “Commission cantonale d’ethique de la Recherche” of Geneva (CCER-2018-00229) and all participants provided written informed consent, signed by their legal guardian.

### Testing procedure

The present sensitivity analysis used a procedure similar to that of a previous study^13^. All subjects were equipped with the CGM marker set^3^ (14 mm) and walked barefoot at a self-selected speed along a 10 m walkway. Marker trajectories were tracked by a 12-camera motion capture system (Oqus7+, Qualisys, Göteborg, Sweden) at a frame rate of 100 Hz. Gait kinematics were processed using a Vicon Plug-in Gait software clone—provided as ‘CGM 1.1’ by the PyCGM2 open-source library—that uses a static trial for calibration^15^.

The analysis used the eight markers required to define one of the lower limbs. The present study only considered the left leg. For the pelvis, we calculated a virtual marker at the midpoint of the posterior iliac spine (SACR, for sacrum), between the right and left anterior iliac spine (RASI and LASI, respectively) markers. Then, we considered the lateral femoral epicondyle (LKNE, for lateral knee), lateral tibial malleolus (LANK, for lateral ankle), and the second metatarsal head of the foot (LTOE, for lateral toe). The medial femoral epicondyle and tibial malleolus markers were not used to calibrate the knee- and ankle-joint centers. However, the femoral and tibial wands were included (LTHI, for left thigh, and LTIB, for left tibia, respectively).

**Figure 1:**
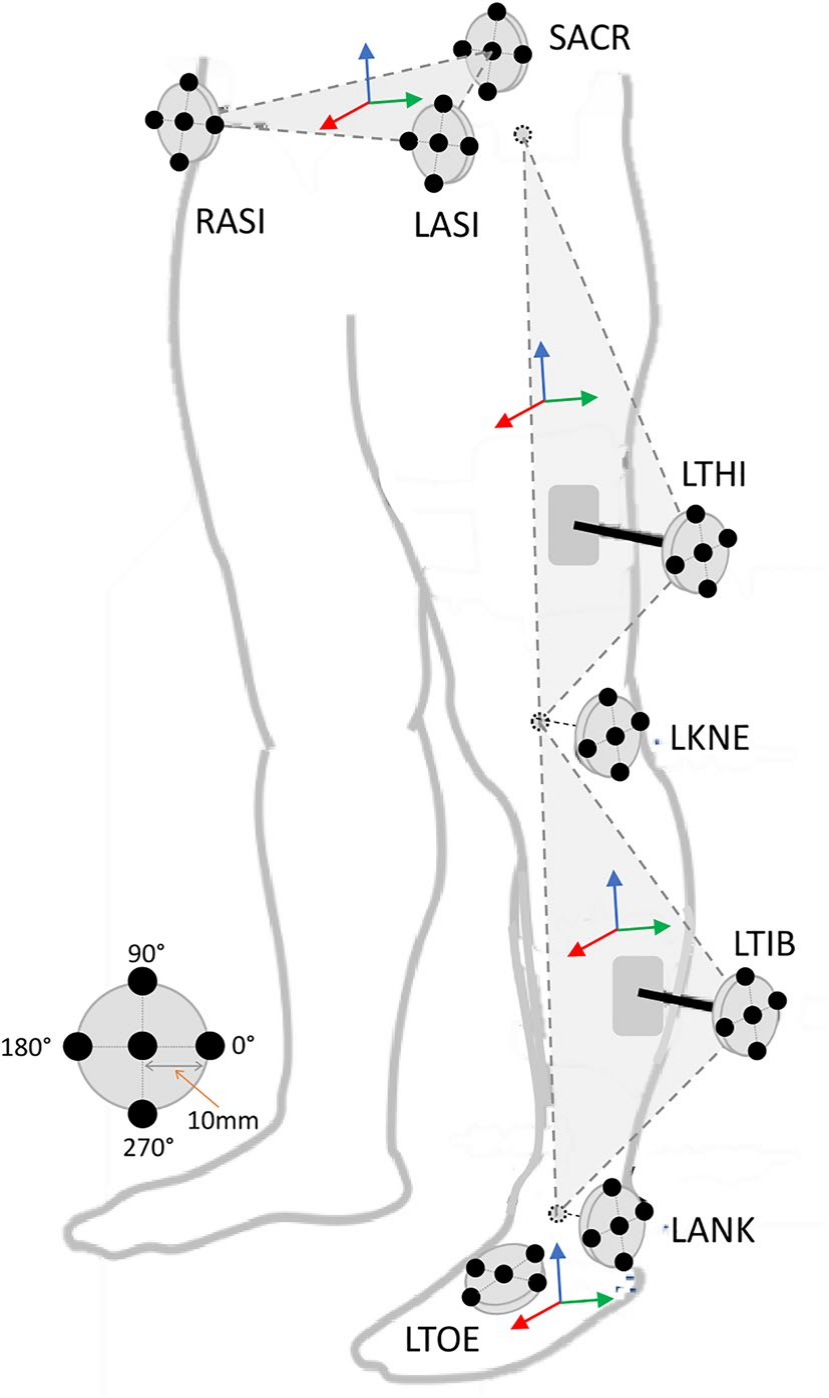
Marker displacement illustration for each marker. Each marker displacement occurred in a defined plane: LASI, RASI, and SACR were displaced in the coronal plane; LTHI, LTIB, LKNE, and LANK were displaced in the sagittal plane; and LTOE was displaced in the transversal plane.

We added a specific offset to the segmental reference frames for each static and dynamic trial, thus creating a new virtual marker and new virtual marker trajectories. These offsets were a ‘displacement’ of 10 mm from each marker’s original position in four different directions (every 90° around the original position) and in their main plane of action. More specifically, as described in Fig. 1, the pelvis markers were displaced in the coronal plane in the medial–lateral (0°, 180°) and proximal–distal (90°, 270°) axes. The foot marker was displaced in the transversal plane in the lateral–medial (0°, 180°) and anterior–posterior (90°, 270°) axes. Finally, the remaining markers (wands on tibia (LTIB) and thigh (LTHI), LANK and LKNE) were displaced in the sagittal plane in the anterior–posterior (0°, 180°) and proximal–distal (90°, 270°) axes. Each marker’s original position was also included, giving us five different virtual positions for each marker. Every possible combination of these marker positions was considered, resulting in 390,625 displacement simulations. This number (n_sim_) was calculated as $n_{sim} = s^{m}$, where *s* represents the number of positions considered for each marker and *m* represents the number of markers considered. For each marker displacement simulation, every other marker position was defined and lower-limb kinematic data were computed together with the GPS. Due to the great computational resources required because of the high number of simulations planned (at around 10 s per simulation, or 45 days of computation per subject with a standard computer), calculations were performed using a high-performance, multi-core computing system suitable for parallel computation. The toolbox used for this study is available at https://gitlab.unige.ch/KLab/multi-marker-misplacement.git.

### Statistical analysis

For each simulation, a root-mean-square deviation (RMSD) angle was calculated between the original kinematics and simulated data kinematics. To better understand the impact of combined marker displacements, we separated the simulations into four categories of angle variability according to their mean RMSD. Therefore, each group’s overall RMSD fell into the angle interval categories of: (1) lower than or equal to 2°; (2) higher than 2° and lower than or equal to 5°; (3) higher than 5° and lower than or equal to 10°; and (4) higher than 10°^8^. The distribution of variability resulting from these displacement simulations was also extracted.

## Results

**Figure 2:**
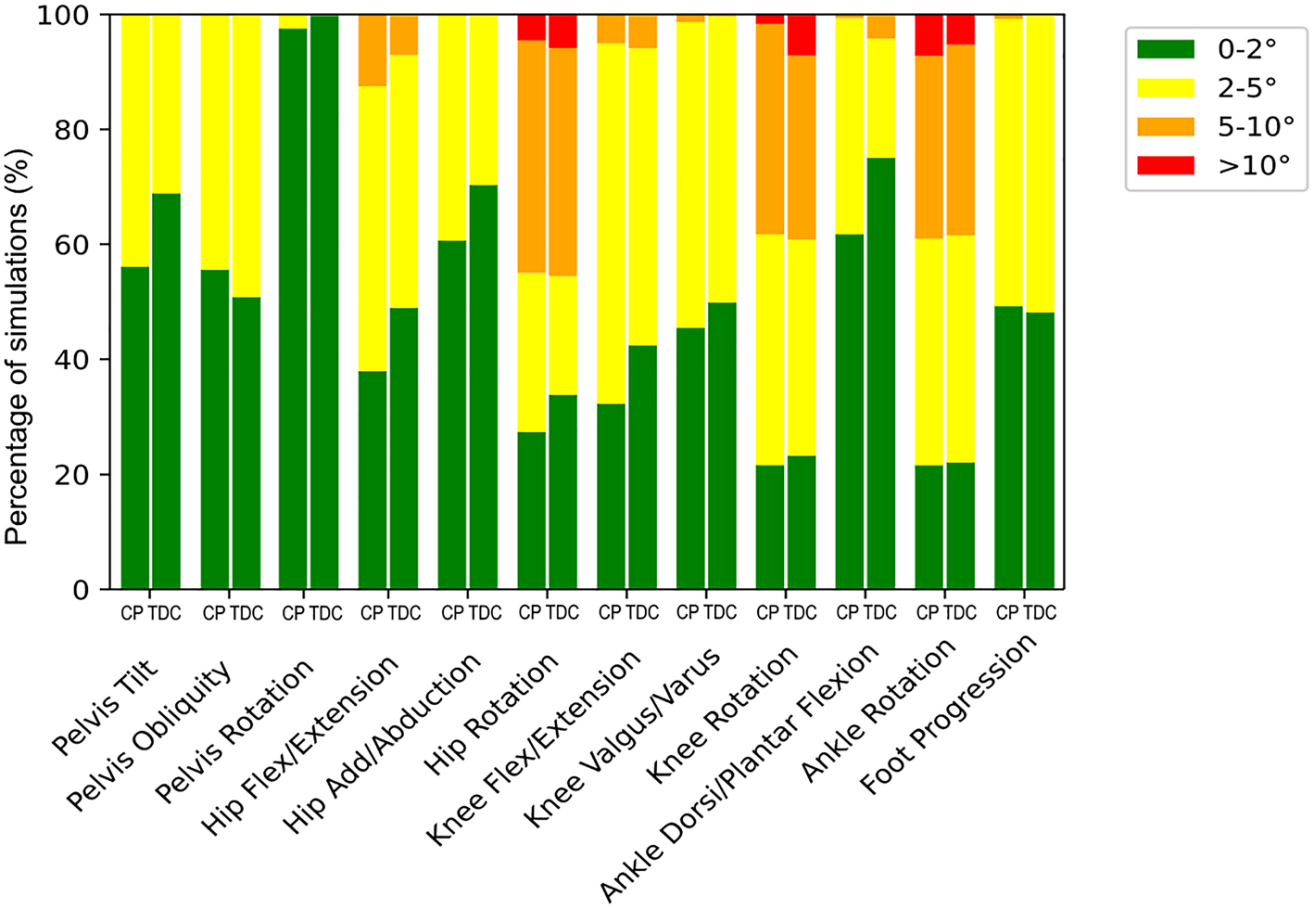
Distribution, in percent, of simulations with RMSDs within the four categories of angle variability (RMSD of 0°–2°, > 2°–5°, > 5°–10° and > 10°) for the two populations (cerebral palsy = CP; typically developed children = TDC).

**Figure 3:**
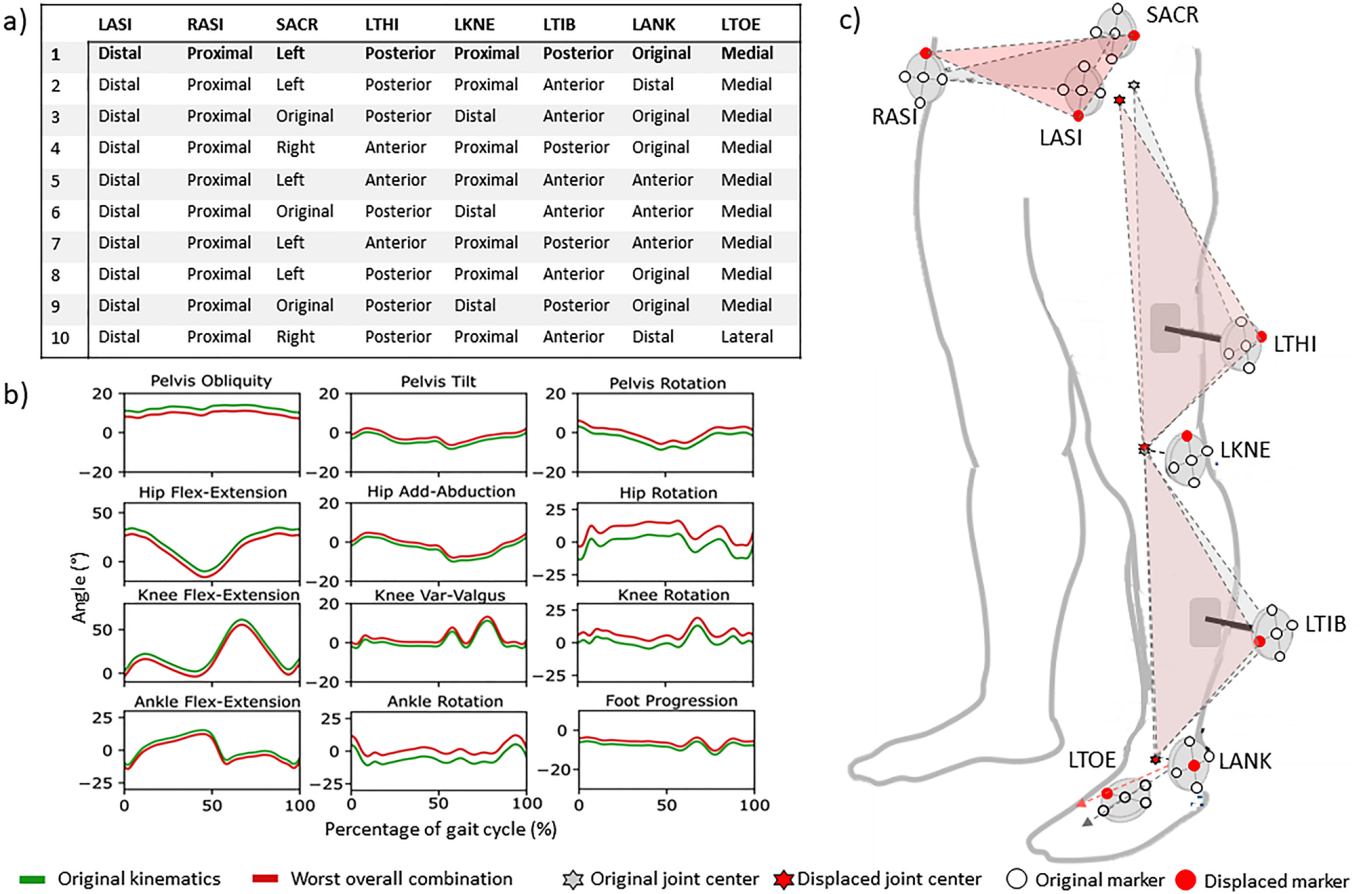
Overall worst-case scenario. (**a**) The 10 simulations resulting in the highest overall RMSD angles in kinematics. Described the direction of displacement of the eight markers for each of the scenarios. (**b**) Kinematics of overall worst-case scenarios (red) plotted against the original (green) kinematics of one CP patient. (**c**) Illustration of impact of worst-case scenario (bold in the table).

Figure 2 illustrates the distributions (in percent) of displacement simulations with RMSDs in each of the four categories of angle variability for both groups of subjects. Multiple marker displacements (8% of all simulations) resulted in hip, knee, and ankle rotations of over 10° of RMSD, and over 40% of all simulations resulted in rotations over 5°. Nearly 10% of hip, knee, and ankle flexion–extension and knee varus/valgus simulations resulted in rotations over 5°. All the displacement simulations on the other joint angles resulted in RMSDs less than 5°. No considerable differences were observed between the two populations. The combinations of displacements resulting in the ten highest overall RMSD are described in Fig. 3a, as well as the kinematics resulting from the worst-case scenario Fig. 3b and its representation on the lower limb definition in comparison with the original marker placement in Fig. 3c. In all the displacement simulations referred, the anterior iliac spines (RASI and LASI) were noted to be displaced in opposite directions in the vertical axis, and the SACR was displaced in the horizontal axis or, in some cases, was not displaced at all. The wands shifted in the anterior–posterior axis; the LKNE was displaced in the proximal–distal axis. Finally, the LANK was displaced either distally, anteriorly, and not at all, whereas the LTOE was displaced in the medial–lateral axis.

**Figure 4:**
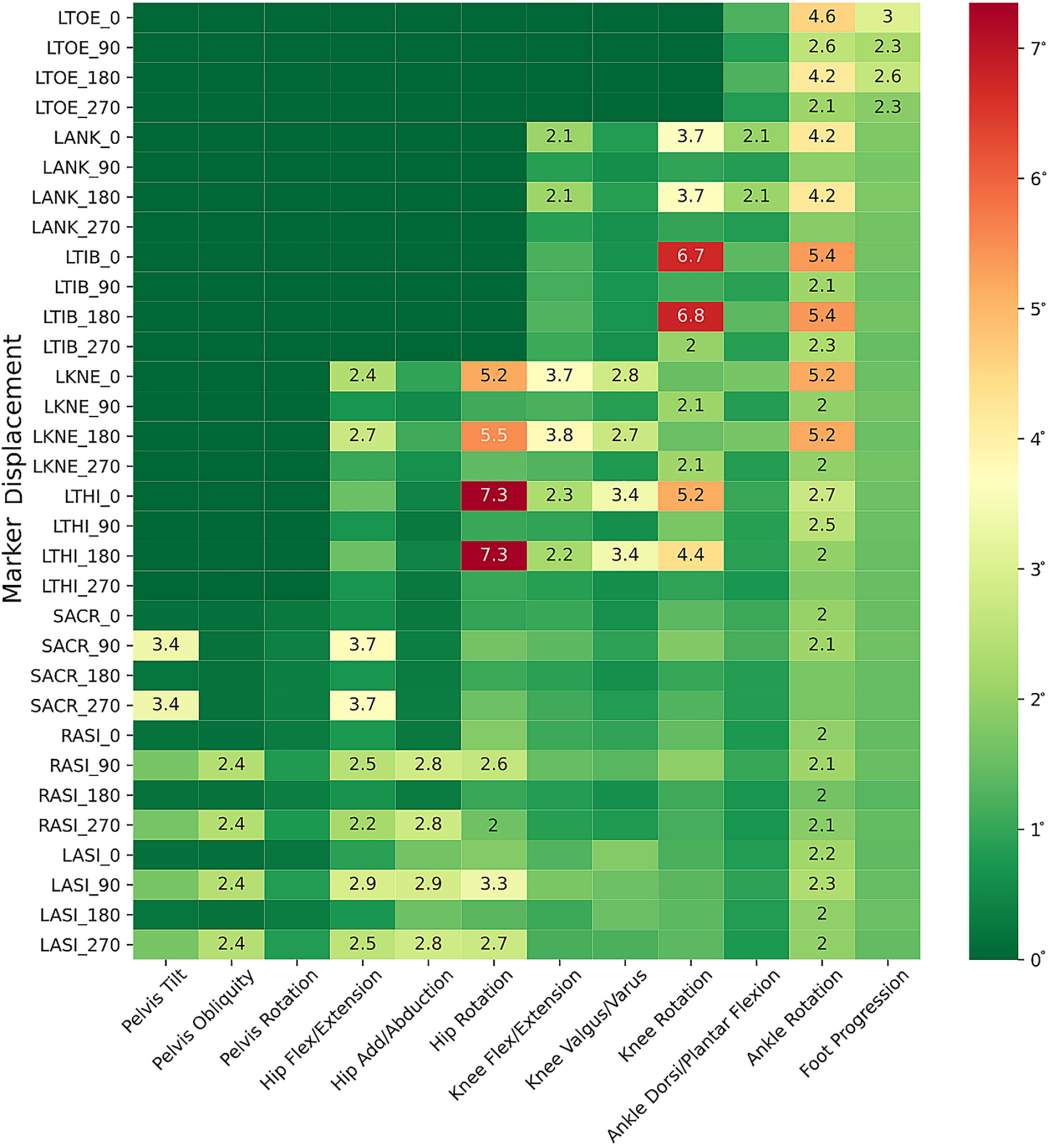
Impact of a single marker displacement on each of the angles across the entire group. Cells only indicate values when the RMSD angle was greater than 2° for the respective marker displaced.

Figure 4 demonstrates the impact of a series of single marker displacements (vertical axis) on different joint kinematics (horizontal axis). Simulated displacements of the thigh and tibia wands (LTHI and LTIB) and the knee marker (LKNE) in the anterior–posterior axis resulted in the highest RMSD of all markers, with RMSD angles for hip, knee, and ankle of over 5° in the transversal plane. The highest mean RMSD angle calculated is relative to the displacement of the femoral wand marker in the anterior–posterior axis, with a hip rotation angle, with a mean RMSD of 7.3° (SD: 1.8°).

**Figure 5:**
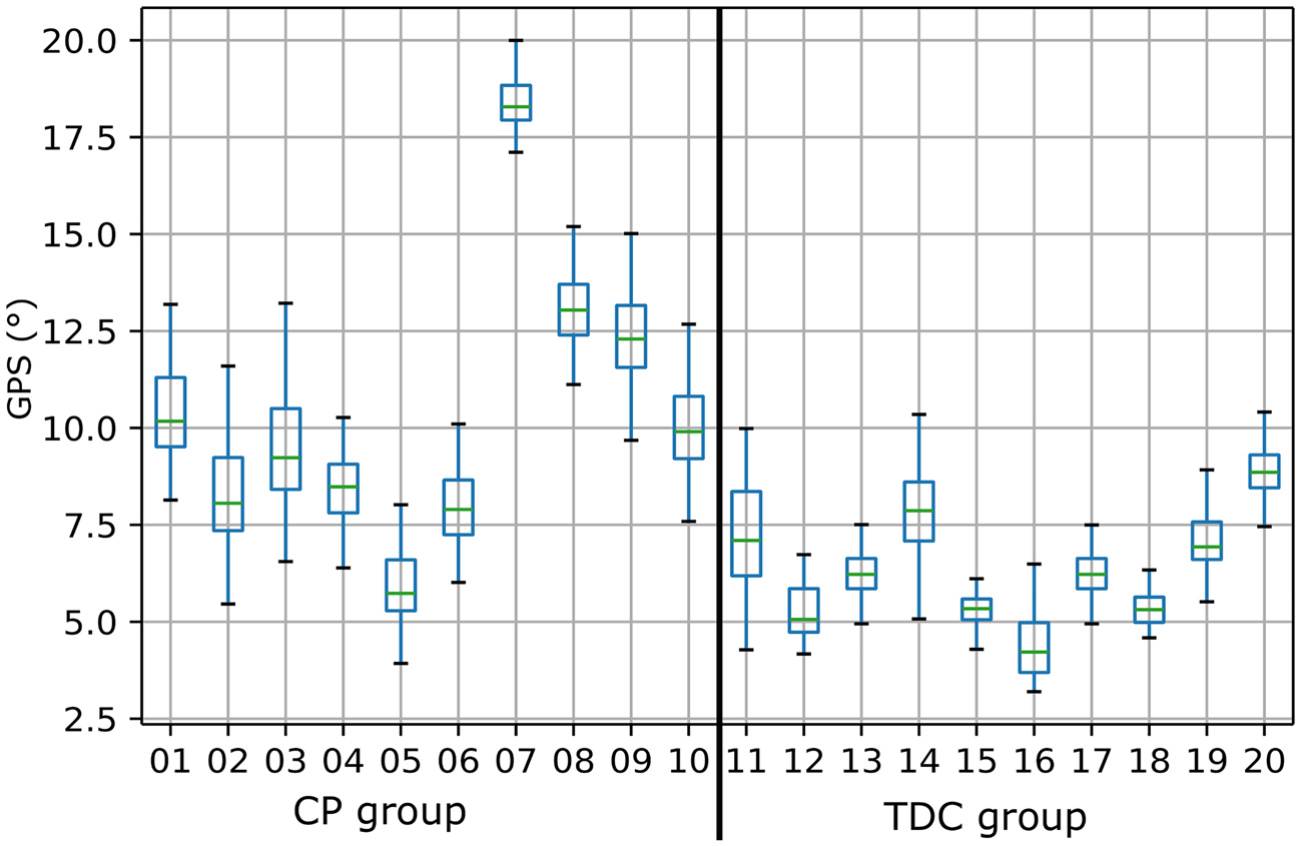
Distribution of Gait Profile Scores for each subject calculated from their simulated displacement data.

**Figure 6:**
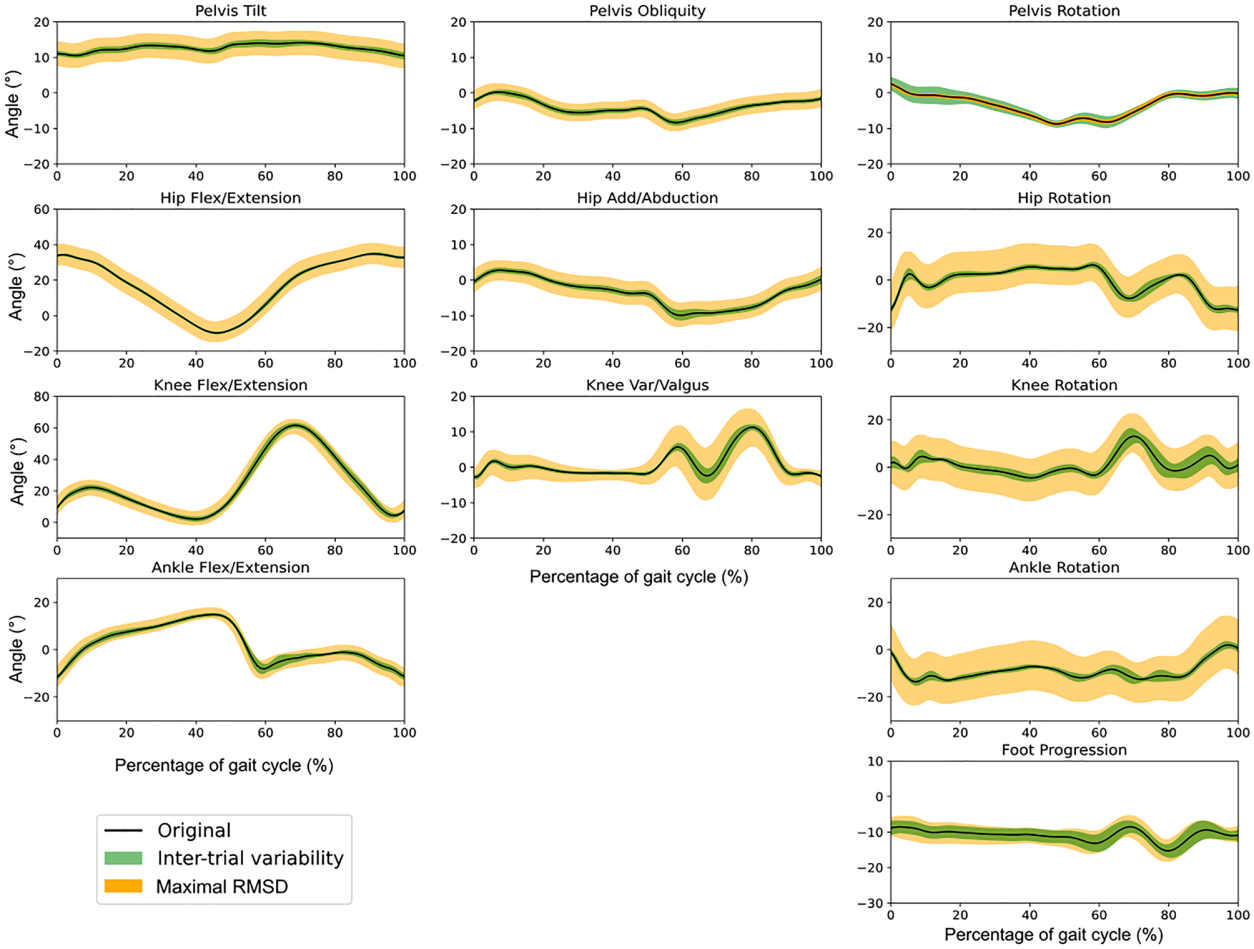
Variability induced by marker displacement. Original kinematics (solid line), inter-trial variability (green corridor), and maximal RMSD angle (yellow corridor) calculated for each point in the gait cycle for a representative child with cerebral palsy.

We also investigated the impact of marker displacement on each subject’s GPS, and their distributions are reported in Fig. 5. The results showed a different variability among the population as the amplitude of scores for each subject varied from 2 to 7 points to its original calculated score. In general, the CP group had higher simulated gait profile scores. Finally, Fig. 6 shows the original kinematics of one representative child with CP together with the corridors of simulated RMSD angles (using the maximum value calculated at each point in the gait cycle). The RMSD angle added by marker displacement was considerably higher than the inter-trial variability, except for the kinematics of pelvis rotation and foot progression angle.

## Discussion

This study’s objective was to evaluate the CGM’s sensitivity to marker placement. We simulated different combinations of markers on the lower-left limb, displaced in different directions by 10 mm. Overall, measurements in the transversal plane demonstrated the greatest sensitivity to marker displacement, whereas the markers displaced in the sagittal plane resulted in the highest RMSD angles in comparison to the original kinematics.

Pelvic kinematics showed very low sensitivity to marker displacement, with all their RMSD angles calculated to within an acceptable limit of 5° and the majority of their simulations resulting in RMSD angles within 2°. For the other joint angles calculated, the transversal plane was the most affected by marker displacement, with about 47% of simulations returning an error over the 5° limit of acceptability. These findings agreed with previous literature reporting that the transversal plane was the least reliable in gait analysis^2,16^.

An analysis of the ten worst-case marker misplacement scenarios allowed us to better understand the effects of a combination of marker displacements on the lower-limb model. For instance, the ‘worst’ marker configuration for the pelvis was calculated for when the anterior iliac spines markers were displaced in opposite directions in the vertical axis and the SACR was displaced in the horizontal axis. With this simulated marker configuration, the pelvis was both tilted and rotated with respect to its original definition. Because the CGM is a hierarchical, anatomical, top-down model, this would be expected to affect the hip-joint centre estimation, hip kinematics, and all the distal joint angles. As illustrated in Fig. 3, the CGM’s thigh and tibia’s flexion–extension axes are defined as orthogonal to the plane connecting the proximal and distal joint centres when the wand is placed along the segment. Thus, those segments’ medial–lateral axes are estimated to be orthogonal to both the flexion–extension and proximal–distal axes. The simulated displacement of the femoral wand (LTHI) in the anterior–posterior axis directly affects the femur’s coronal plane, thus altering the flexion–extension axis and the medial–lateral axis. As a consequence, the kinematics of the hip and knee joints will be directly affected, as will the knee joint centre that is defined along the femur’s medial–lateral axis (in the absence of the medial femoral epicondyle marker). A similar impact was noted for the tibia. Finally, the medial displacement of the LTOE marker was responsible for a rotation of the foot’s angle with respect to the direction of walking and for an impact on the foot progression angle.

Regarding displacements of individual markers, displacements of the thigh and tibia wands and the knee marker in the anterior–posterior axis had the largest calculated impact on kinematics, all with an RMSD angle of over 5° in the transversal plane (Fig. 4). These findings confirmed previous results demonstrating the knee marker’s high impact in the anterior–posterior axis in the transversal plane but it’s very low impact when displaced in the proximal–distal axis^12,13^. Even though some studies have reported improvements in calibration methods, such as the Knee Alignment Device, marker placement reproducibility and reliability remains the CGM’s most significant limitation^17^. The CGM’s high sensitivity to wand orientation is even more critical as the lack of an anatomical landmark makes its placement somewhat subjective. Current user manual specifications for wand placement are simply, “Adjust the position of the marker so that it lies in the plane that contains the hip and knee-joint centers and the knee flexion/extension axis”^18^.

The CGM is characterized by a hierarchical, anatomical, top-down approach; therefore, a displaced marker affects the kinematics of every joint located distally to the anatomical segment containing that marker and the joint most proximal to it^15^. Additionally, the slight impact that we calculated on the foot progression angle demonstrates that without the medial markers of the knee and ankle, defining the joint centers is affected by multiple marker displacements. Thus, an error in the placement of the knee-joint center marker impacts the definition of the ankle-joint center and consequently the foot progression angle. Overall, the calculated impact of a displaced marker could be noted in the two simulated displacements in opposite anatomical directions.

Gait scores, like the GPS, are very good at classifying a patient’s gait by comparing it with a reference database of a general asymptomatic population. As the calculation uses kinematic data, the variability noted because of marker displacement also introduces variability into the final gait classification and thus may also have a considerable impact on gait data interpretation. We therefore investigated the impact of marker displacement on overall gait scores. Marker displacement in one leg resulted in GPS variations of up to 7°. This is comparatively much greater than the 1.6° rated as the minimal variation of clinical significance^19^. As the GPS is calculated using the kinematics of both lower limbs, the variation expected if our simulations were applied to both sides would be even higher.

The impact of the variability of marker placement on our simulated gait kinematics is shown in Fig. 5 by the corridors of maximal RMSD angle calculated per frame in the gait cycle added around one subject’s original curve. We note that the error can be defined by an overall offset added to the original data. This finding agrees with previous results reporting that the impact of errors on axis definition was more like an offset to the kinematics than a change in their overall pattern^4^. Such results may be useful for estimating the expected variability in kinematics when considering expected marker placement variability. To evaluate the impact of marker misplacement more accurately, our results could be used in combination with those from studies reporting on the precision of marker placement, such as Della Croce et al.^11^. Thus, the magnitude of each marker’s misplacement would be defined based on experimentally observed error.

Considering the overall results provided within this study, different solutions can be proposed to mediate the displacement of the markers. First, anatomical landmark identification should be followed carefully and with good training of the responsible evaluators. The guidelines used for marker placement in our data are recommended^14^. Secondly, the refered evaluator should have additional attention to the markers and directions which have a large impact on the kinematics, as demonstrated in the Fig. 4. In order to solve the high sensitivity observed on the wand, lateral femoral epicondyle and lateral tibial malleolus to anterior–posterior misplacement, Knee Alignment Device or the medial femoral and tibial markers could be a solution but specific studies are required to validate the possible solution^5,20^. Thirdly, in patients who have undergone 3D imaging, a fusion between medical imaging and motion capture system could limit the marker misplacement but seems difficult to apply to all patients who performed a clinical gait analysis^21^.

The present study had some limitations. Firstly, the lack of literature regarding gait analysis’ sensitivity to marker placement makes comparisons with our results difficult. Secondly, marker displacement was done virtually, so the effects of soft tissue artifacts could not be considered. Different marker displacement distances and axes could also induce different soft tissue artifacts^22,23^. Moreover, our reference marker placements cannot be considered as ‘true’ references as they too were subject to the uncertainty of marker placement. We only applied displacements of 10 mm in only four directions, although that distance was defined according to Della Croce’s results and to serve as a potential standard reference for future comparisons^11^. Finally, the enormous amount of simulations required to compute every potential combination of marker displacement for the twenty subjects required enormous computing time. This imposed limits on the testing of numerous displacement distances and directions, as previously reported for single-marker displacements^13^.

To conclude, we performed a very extensive sensitivity analysis combining 390,625 simulated marker placements. We successfully identified the most sensitive angles contributing to an overall marker displacement simulation measurement and quantified the RMSD angles associated with the displacements of the different lower-limb markers. We also identified and analyzed simulated worst-case marker displacement scenarios. Additionally, we reported on which markers and which axes caused the greatest variability in the angles measured. Greater accuracy in the placement of thigh and tibia wands (or markers) and lateral femoral epicondyle markers in the anterior–posterior axis are required to improve the reliability of gait analysis using the GCM.

## References

[1] Armand S, Decoulon G, Bonnefoy-Mazure A (2016). Gait analysis in children with cerebral palsy. EFORT Open Rev..

[2] Wren TL, Gorton GE, Õunpuu S, Tucker CA (2011). Efficacy of clinical gait analysis: A systematic review. Gait Posture.

[3] Davis RB, Õunpuu S, Gage JR, Tyburski D (1991). A gait analysis data collection and reduction technique. Hum. Mov. Sci..

[4] Kadaba MP (1989). Repeatability of kinematic, kinetic, and electromyographic data in normal adult gait. J. Orthop. Res..

[5] Baker R, Leboeuf F, Reay J, Sangeux M (2016). The conventional gait model: The success and limitations. Handb. Hum. Motion.

[6] Chia K, Sangeux M (2017). Quantifying sources of variability in gait analysis. Gait Posture.

[7] Gorton GE, Hebert DA, Gannotti ME (2009). Assessment of the kinematic variability among 12 motion analysis laboratories. Gait Posture.

[8] McGinley JL, Baker R, Wolfe R, Morris ME (2009). The reliability of three-dimensional kinematic gait measurements: A systematic review. Gait Posture.

[9] Schwartz MH, Rozumalski A (2008). The gait deviation index: A new comprehensive index of gait pathology. Gait Posture.

[10] Baker R (2009). The gait profile score and movement analysis profile. Gait Posture.

[11] DellaCroce U, Cappozzo A, Kerrigan DC (1999). Pelvis and lower limb anatomical landmark calibration precision and its propagation to bone geometry and joint angles. Med. Biol. Eng. Comput..

[12] McFadden C, Daniels K, Strike S (2020). The sensitivity of joint kinematics and kinetics to marker placement during a change of direction task. J. Biomech..

[13] Fonseca M, Gasparutto X, Leboeuf F, Dumas R, Armand S (2020). Impact of knee marker misplacement on gait kinematics of children with cerebral palsy using the Conventional Gait Model—A sensitivity study. PLoS ONE.

[14] Van Sint Jan S (2007). Color Atlas of Skeletal Landmark Definitions: Guidelines for Reproducible Manual and Virtual Palpations.

[15] Leboeuf F (2019). The conventional gait model, an open-source implementation that reproduces the past but prepares for the future. Gait Posture.

[16] Schwartz MH, Trost JP, Wervey RA (2004). Measurement and management of errors in quantitative gait data. Gait Posture.

[17] Baker R, Leboeuf F, Reay J, Sangeux M (2017). The conventional gait model: The success and limitations. Handb. Hum. Motion.

[18] Fellinger, M., Passler, J. & Seggl, W. Plug-in gait reference guide. *Hum. Nonhum. Bone Identif.* 227–246 (2010).

[19] Baker R (2012). The minimal clinically important difference for the Gait Profile Score. Gait Posture.

[20] Motion Lab Systems, I. Knee Alignment Device: User Manual (2011).

[21] Gasparutto X, Sancisi N, Jacquelin E, Parenti-Castelli V, Dumas R (2015). Validation of a multi-body optimization with knee kinematic models including ligament constraints. J. Biomech..

[22] Sangeux M, Barré A, Aminian K (2017). Evaluation of knee functional calibration with and without the effect of soft tissue artefact. J. Biomech..

[23] Barré A, Jolles BM, Theumann N, Aminian K (2015). Soft tissue artifact distribution on lower limbs during treadmill gait: Influence of skin markers’ location on cluster design. J. Biomech..

